# Managing Diabetes in Patients Hospitalized in Internal Medicine Units

**DOI:** 10.5041/RMMJ.10334

**Published:** 2018-04-19

**Authors:** Irit Hochberg

**Affiliations:** Institute of Endocrinology, Diabetes and Metabolism, Rambam Health Care Campus, Haifa, Israel

**Keywords:** Diabetes, hospital, hyperglycemia, insulin

## Abstract

Diabetes and hyperglycemia are present in over one-third of inpatients in internal medicine units and are associated with worse prognosis in multiple morbidities. Treatment of inpatient hyperglycemia is usually with basal bolus insulin in a dose calculated by the patient’s weight, with lower doses recommended in patients who are at a higher risk for hypoglycemia. Other antihyperglycemic medications and insulin regimens can be used in selected patients. There are no adequately powered studies on the effect of improving glycemic control on hospitalization outcomes in non-critically ill patients in internal medicine units, and in most patients a modest glucose target of 140–180 mg/dL is recommended. A structured discharge plan should intensify antihyperglycemic treatment as needed and include an outpatient follow-up appointment shortly after discharge.

## THE PREVALENCE OF DIABETES IN INTERNAL MEDICINE UNITS

Diabetes and hyperglycemia are present in up to 45% of inpatients in internal medicine units.^1^–^3^ In the majority of cases hyperglycemia is not the primary reason for hospitalization, but in many cases it contributes to the conditions that led to hospitalization, such as cardiovascular events, heart failure, renal failure, and infections. These diagnoses are all more prevalent in patients with diabetes, and the pathophysiology through which hyperglycemia causes or worsens these conditions has been well characterized.

Hyperglycemia is an unfavorable prognostic sign in patients admitted with a multitude of medical diagnoses common in patients hospitalized in internal medicine wards, including, but not limited to, pneumonia,^4^ stroke,^5^ myocardial infarction,^6^ and chronic obstructive pulmonary disease exacerbation.^7^

## INSULIN TREATMENT IN INTERNAL MEDICINE UNITS

Subcutaneous insulin is the standard of care in most non-critical hospitalized patients.^8^,^9^ It is safe, there are no contraindications, and it has a rapid effect, which enables improvement in glycemic control in a few days. The flexibility conferred by insulin enables the nurse to take into account the level of glucose and the nutritional state of the patient for each insulin injection, leading to a safe and rapid improvement of hyperglycemia. The insulin regimen recommended by all guidelines for treating inpatient hyperglycemia in non-critical settings is basal bolus correction.^8^ An insulin order set consists of a once-daily subcutaneous basal insulin and thrice daily prandial rapid-acting insulin which is corrected according to glucose levels. A sample insulin order protocol appears in Box 1. The safety of this protocol in internal medicine units and its superiority over the now-extinct sliding scale insulin regimen used in the past have been demonstrated in the RABBIT-2 trial.^10^ Another safe and effective protocol, basal plus, consists of a once daily subcutaneous basal insulin and thrice daily correction.^11^ Insulin mixes lead to significantly more hypoglycemia episodes compared to basal bolus insulin in medical and surgical wards^12^ and should usually be avoided.

Box 1 **Example of a Pragmatic Simplified Insulin Order Set in Internal Medicine**

Glucose targets in most patients in internal medicine are 90–180 mg/dL. Targets can be lower in selected stable patients.

Discontinue oral and non-insulin injectable diabetes drugs in patients who have either:

- Renal/hemodynamic instability
- Unstable nutrition (intermittent NPO [nil per os, or Nothing by mouth] for tests/procedures or secondary to illness)
- Recurrent glucose measurements over 180 mg/dL

Initiate basal bolus insulin in patients with recurrent glucose measurements over 180 mg/dL. Calculate the total daily dose according to the patient weight and:

- 0.3 U × kg of body weight in patients over 70 years or glomerular filtration rate <60 mL/min
- 0.5 U × kg of body weight per day for patients not meeting the above criteria

Divide the total daily insulin dose as:

- 50% basal insulin (glargine/detemir) once daily
- 50% prandial insulin (lispro/aspart/glulisine) divided to thrice daily

In a patient *that is eating* (including enteral feeding), give prandial insulin and adjust dose by adding/subtracting correction insulin according to the preprandial glucose measurement.

In a patient *that is not eating*, reduce basal insulin dose by 20%, omit prandial insulin, and give correction insulin thrice daily according to the results of glucose measurement.

**Table:** 

Glucose (mg/dL)	Insulin Correction (added to prandial insulin or given as is in a patient that is not eating)
Less than 70	No prandial insulin
Less than 90	Reduce 4 units
90–180	No correction
181–250	Add 2 units
251–300	Add 4 units
301–350	Add 6 units
351–400	Add 8 units
Over 400	Report to physician

Insulin dose adjustment should be considered every morning:

- If glucose measurements are persistently above 180 mg/dL, increase insulin dose by 10%–20%
- If there are glucose measurements less than 90 mg/dL, decrease insulin dose by 10%–20%

## TREATMENT WITH NON-INSULIN ANTIHYPERGLYCEMIC MEDICATIONS

There are no high-quality studies establishing the safety of most non-insulin antihyperglycemic medications in the hospital setting, and there are safety concerns in using them in many hospitalized patients due to an increased risk for worsening of heart failure, renal failure, or anemia. Renal failure may lead to accumulation of medications which can increase the risk of dangerous side effects including hypoglycemia and lactic acidosis. In addition, taking some of these medications in the very common situations where nutrition is halted increases the risk of hypoglycemia, dehydration, and ketoacidosis. On the other hand, in patients whose glucose measurements are at target level, with stable nutrition and without new contraindications, many clinical guidelines allow continuance of preadmission medications as this reduces the burden on the nursing staff and prevents deterioration of glycemic control.

Retrospective studies have reported a high rate of hypoglycemia in inpatients treated with sulfonylureas.^13^,^14^ A dipeptidyl peptidase 4 inhibitor with basal insulin has shown non-inferiority in glycemic control and safety compared to basal bolus insulin in patients hospitalized in medical wards.^15^ A glucagon-like peptide-1 agonist has shown non-inferiority in glycemic control in intensive care unit (ICU) patients, most of which did not have preadmission diabetes, but an increase in nausea.^16^ There are no specific studies on use of metformin, sodium-glucose cotransporter-2 inhibitors, pioglitazone, or acarbose in hospitalized patients, but experience shows that in selected patients they are safe to use. While in the past guidelines had recommended temporary interruption of metformin treatment before exposure to contrast media, most current guidelines recommend such interruption only in patients with chronic renal failure (with a cutoff of glomerular filtration rate of <60 or <45 mL/min per 1.73 m^2^).^17^–^19^

## EFFECT OF GLYCEMIC CONTROL IN INTERNAL MEDICINE UNITS ON HOSPITALIZATION OUTCOMES

There are no large randomized controlled studies on significance of improved glycemic control on hospitalization outcomes in hospitalized medical patients outside the intensive care setting.^20^ Nevertheless there are studies on effect of glycemic control on outcomes in some specific medical conditions commonly found in medical wards.

### Ischemic Heart Disease

Hyperglycemia is associated with a worse prognosis in patients with myocardial infarction.^6^ A single randomized control study has shown that improving glycemic control in patients with an acute myocardial infarction improves survival.^21^ A second study which is problematic to interpret as it did not achieve a long-term difference in glycemic control between study groups found no difference in survival.^22^

### Stroke

Glucose treatment targets in stroke patients are not clear, and current studies have not found benefit or harm in reducing glucose levels for the first 24 hours using intravenous insulin in stroke patients.^23^ On the other hand, none of the studies was powered to detect an effect on outcomes, and an ongoing larger study is being conducted.^5^ The current recommendation is like in most hospitalized patients to aim for glucose levels under 180 mg/dL.

### Renal Failure

Insulin is eliminated by the kidneys, and patients with renal failure have a higher risk of developing hypoglycemia.^24^ Therefore treatment guidelines recommend lower insulin doses for patients with renal failure.^25^ This recommendation is supported by results of an inpatient randomized control study, that in patients with a glomerular filtration rate ≤45 mL/min/1.73 m^2^ a lower total daily dose of insulin (0.25 units/kg) reduced the risk of hypoglycemia compared to a standard dose (0.5 units/kg ) without hindering glucose control.^26^

### Patients with Advanced Age

Advanced age is another risk factor for hypoglycemia,^27^,^28^ and lower insulin doses are usually used in elderly patients. Nevertheless, there is evidence that a basal bolus regimen is safe and effective in geriatric patients.^29^

## GLYCEMIC TARGETS IN INTERNAL MEDICINE UNITS

Studies in intensive care have found harm in intensive insulin regimens aiming for euglycemia,^30^ most likely secondary to increased risk of hypoglycemia.^31^ Although there are no similar large randomized studies powered to prove a benefit in improving glycemia control in internal medicine units, the current guidelines recommend aiming for a glucose target of 140–180 mg/dL in most non-critically ill and critically ill hospitalized patients.^30^

## HYPOGLYCEMIA

Avoiding hypoglycemia is a main concern of physicians and nurses when treating hyperglycemia in hospitalized patients. Data from randomized controlled trials indicate a prevalence of hypoglycemia between 5% and 32% in non-ICU patients treated with subcutaneous insulin.^28^ Older age, renal failure, and higher doses of insulin are associated with higher risk for hypoglycemia, and therefore renal function and age should be taken into account in decisions on insulin dosing.^8^ A meta-analysis of randomized controlled studies of basal bolus insulin compared to sliding scale insulin demonstrated that mild hypoglycemia events (defined as glucose levels under 70 mg/dL and over 40 mg/dL) occur in 23% of medical patients, more commonly than in surgical patients. There was no difference in the occurrence of severe hypoglycemia events (defined as glucose under 40 mg/dL) and no increase in mortality in patients with insulin-induced hypoglycemia.^28^

## HOSPITAL DISCHARGE OF PATIENTS WITH HYPERGLYCEMIA

Because discharge orders are based on prior glycemic control as reflected in admission hemoglobin A1c (HbA1c) levels, guidelines recommend performing an HbA1c test on all patients with diabetes or hyperglycemia admitted to the hospital if there are no up-to-date results from the prior 3 months. In patients with adequate glycemic control prior to hospital admission, as determined by a glycated hemoglobin at target (usually under 7%, but higher for patients that are older, have a shorter life expectancy, or with a higher burden of comorbidities), it is safe to return upon hospital discharge to the previous antihyperglycemic treatment.^32^ In patients with inadequate prior glycemic control, intensification of treatment is recommended.^8^ Adding basal insulin at 50% of hospital daily dose to the preadmission antihyperglycemic regimen in patients with HbA1c between 7% and 9%, and either basal insulin or basal bolus insulin at 80% of inpatient dose in those with HbA1c>9%, is safe and effective in improving glycemic control.^32^ There are no specific studies on treatment intensification with other antihyperglycemic medications in the setting of hospital discharge, but there are many non-insulin options that can improve glycemic control and may have additional benefits over insulin, including oral delivery, less hypoglycemia risk, and even reduction of cardiovascular events and diabetic nephropathy in selected patients.^33^ Regardless of which treatment was added, a structured discharge plan is essential, including an outpatient follow-up visit with the primary care provider, endocrinologist, or diabetes nurse specialist/educator within 1 month of discharge for all patients that had hyperglycemia in the hospital. An appointment within 1–4 weeks of discharge is recommended if glycemic medications are changed or glucose control is not optimal at dis-charge.^9^ See Box 2 for a sample discharge protocol.

Box 2 **Example of a Hospital Discharge Protocol**

Decide whether diabetes treatment needs to be changed at discharge:

- HbA1c is within target and there are no contraindications — resume preadmission treatment
- HbA1c above target or contraindications to preadmission treatment — modify therapy:
  ➢ HbA1c >9%: add basal insulin to preadmission treatment or switch to basal bolus insulin
  ➢ HbA1c above target but <9%, or contraindications to preadmission treatment — start/add another therapy (oral antidiabetes medications/non-insulin injectable antidiabetes medication/basal insulin)

Ensure the patient has the equipment and prescriptions needed for home treatment (glucometer, strips, lancets, needles)

The patient must receive oral and written explanations on:

- When and how to take medications
- When and how to measure glucose
- How to recognize, treat, and prevent hypoglycemia
- Basic diet planning and instruction for follow-up with a dietician
  ➢ Follow-up visit with family physician/diabetes specialist/nurse specialist/diabetes educator within 1–4 weeks after discharge (urgency depends on patient characteristics and complexity of the treatment)

## CONCLUSION

Hyperglycemia is common in internal medicine units, and the recommended treatment is basal bolus insulin. Certain patients are at higher risk for hypoglycemia and should receive lower insulin doses. Despite lack of strong evidence for improvement in hospitalization outcomes, glucose targets in most patients should be 140–180 mg/dL. A structured discharge plan should intensify antihyperglycemic treatment as needed and include an outpatient follow-up appointment shortly after discharge.

## Abbreviations

ICU: intensive care unit
HbA1c: hemoglobin A1c.
